# Biology and Biotechnology of Follicle Development

**DOI:** 10.1100/2012/938138

**Published:** 2012-05-22

**Authors:** Gustavo Adolfo Palma, Martin Eduardo Argañaraz, Antonio Daniel Barrera, Daniela Rodler, Adrian Ángel Mutto, Fred Sinowatz

**Affiliations:** ^1^CICyTTP, CONICET, Dr. Materi y España s/n Entre Ríos, 3105 Diamante, Argentina; ^2^Laboratory of Biotechnology of Reproduction, Department of Animal Production, National University of Santiago del Estero, 4200 Santiago del Estero, Argentina; ^3^Instituto de Biología, Facultad de Bioquímica, Universidad Nacional de Tucumán, Ayacucho 491, 4000 San Miguel de Tucumán, Argentina; ^4^Institute of Anatomy, Histology and Embryology, Department of Veterinary Sciences, University of Munich, Veterinaerstrasse 13, 80539 Munich, Germany; ^5^Biotecnología Aplicadas a la Reproducción Animal en IIB-INTECH, Camino Laguna Km 6 B7130IWA Chascomús, Argentina

## Abstract

Growth and development of ovarian follicles require a series of coordinated events that induce morphological and functional changes within the follicle, leading to cell differentiation and oocyte development. The preantral early antral follicle transition is the stage of follicular development during which gonadotropin dependence is obtained and the progression into growing or atresia of the follicle is made. Follicular growth during this period is tightly regulated by oocyte-granulosatheca cell interactions. A cluster of early expressed genes is required for normal folliculogenesis. Granulosa cell factors stimulate the recruitment of theca cells from cortical stromal cells. Thecal factors promote granulosa cell proliferation and suppress granulosa cell apoptosis. Cell-cell and cell-extracellular matrix interactions influence the production of growth factors in the different follicular compartments (oocyte, granulosa, and theca cells). Several autocrine and paracrine factors are involved in follicular growth and differentiation; their activity is present even at the time of ovulation, decreasing the gap junction communication, and stimulating the theca cell proliferation. In addition, the identification of the factors that promote follicular growth from the preantral stage to the small antral stage may provide important information for the identification for assisted reproduction techniques.

## 1. Introduction

The study of *in vitro *development of oocytes gives important information on the abilities which acquire oocytes during their early follicular stage *in vivo*.

Recently it was demonstrated that the inherently high variation in follicle numbers during follicular waves is also associated with significant alterations in intrafollicular estradiol production, which is the hallmark for follicular function, and expression of key genes important for differentiation, function, and survival of thecal, granulosal, and cumulus cells in the largest three follicles growing during follicular waves [1].

The follicle is an ovarian structure with two major functions, namely, the production of hormones and growth of oocytes capable of being fertilized. These functions are carried out by antral follicles which posses an inner wall of granulosa cells that rest on a distinct basal lamina. This specialized extracellular matrix separates the epithelial layer from the connective tissue and affects proliferation and differentiation of the granulosa cells [2, 3].

**Figure 1:**
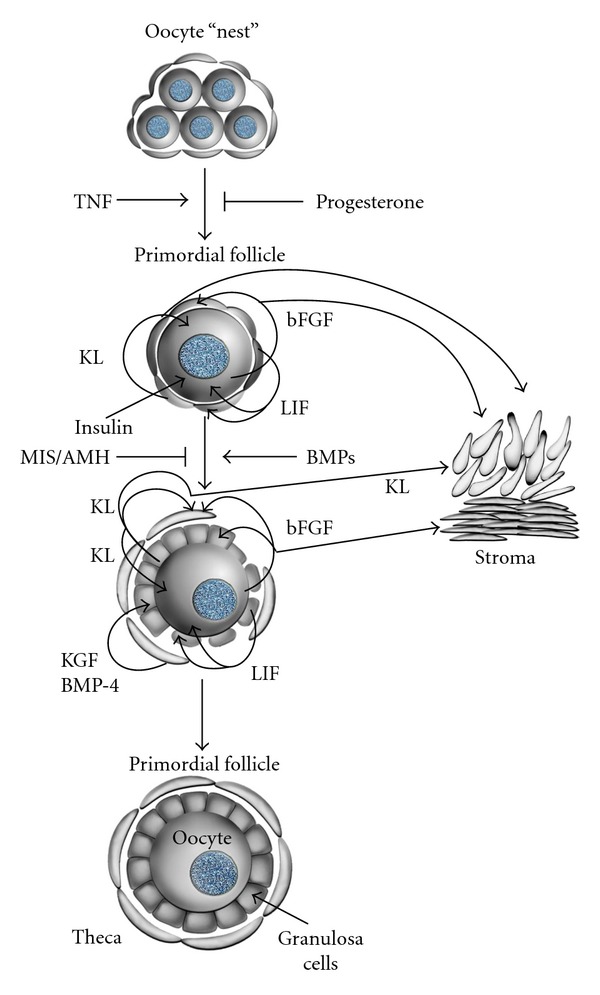
Cell interactions in primordial follicles [3, 6]. Proposed cell interactions in the development of primordial follicles and the effect of the growth factors. Cell-cell interactions are mediated by tumor necrosis factor-alpha (TNF*α*), Kit ligand (KL), basic fibroblast growth (bFGF), leukemia inhibitory factor (LIF), bone morphogenetic protein-4 (BMP-4), keratinocyte growth factor (KGF), insulin, and anti-Mullerian hormone (AMH). With permission from M. Skinner.

Oocytes of mammals develop and reach ovulatory maturity inside the follicles. A follicle is made up by the oocyte covered by pregranulosa or granulosa cells (Figure 1). Ovarian development in the embryo starts between 3 and 6 weeks after conception, a period in which numerous cellular events take place, such as massive colonization of the ovary with mesonephric cells, which are regarded as one of the precursors of the follicle cells [4, 5], migration of the primordial germ cells into the genital ridge, gonadal sex differentiation, mitosis, and apoptosis of the germ-cells. Follicular development and atresia already begin during fetal life in domestic animals and primates.

The process of germ cell formation includes several stages:

- generation of the primordial germ cells (PGCs) in the yolk sac,
- migration of PGCs to the genital ridge,
- colonization of the gonads by the PGCs,
- differentiation of the PGCs to oogonia,
- oogonial proliferation,
- meiotic initiation,
- arrest in the diplotene stage of meiotic prophase.

In this paper we put the focus on the modifications, which occur during the development of primordial and primary follicles, the molecular mechanisms of cell-cell and cell-extracellular matrix interaction during follicular development, and on the factors, which induce the acquisition of meiotic competence during oocyte development.

## 2. Primordial and Primary Development of the Follicle

At present, the oocyte is considered to play an important role in follicular organization during the processes leading to ovulation. It is assumed that the oocyte controls the proliferation of granulosa cells and later their differentiation into steroids and protein-secreting cells. On the other hand, granulosa cells are indispensable for oocyte growth, differentiation, meiosis, cytoplasmic maturation, and control of transcriptional activity within the oocyte [15]. When the oocyte reaches a certain size threshold, it secretes factors that inhibit the ability of granulosa cells to promote oocyte growth [16, 17]. This indicates that the oocyte determines not only the growth of the follicle but indirectly also its own growth.

The assembly of primordial follicles takes place during fetal life in the human and bovine species. It begins with an oocyte incompletely surrounded by flattened cells, called pregranulosa cells (Figure 1). Primordial germ-cells (PGCs), the predecessors of the oocytes, develop during gastrulation, when the embryo differentiates into the germ cell layers ectoderm, mesoderm, and endoderm [18]. These cells first become recognizable in the posterior rim of the embryonic disc at gastrulation. From here they move into the newly formed mesoderm and endoderm. A few days later, the PGCs are found in the visceral mesoderm and surrounding the yolk sac and the allantois [5], probably to protect them from the differentiation signals driving gastrulation within the embryo proper. Here they proliferate and migrate via the primitive mesentery into the still undifferentiated but developing gonad. During their migration, the PGC can be recognized by the use of special staining techniques, such as those for alkaline phosphatase activity and expression of the transcription factor OCT4, which plays a role in maintaining cellular pluripotency in the embryo. Whether this migration is caused by the PGCs own active movements by its cytoskeleton or rather as consequence of the progressive pressure caused by the increasing movements of the adjacent tissue is still a matter of debate. At the beginning of the active phase of their migration, PGCs modify their morphological and biochemical characteristics, since they acquire an elongated shape and increase markedly the alkaline phosphatase activity.

During and after their migration, the PGCs proliferate by mitoses. In the female, germ cells become surrounded by somatic cells, probably derived from the surface epithelium of the developing gonads and/or from cells invading from the mesonephros. When the PGCs are now enclosed by presumptive follicle cells they are referred to as oogonia. Although still not proven, meiosis-stimulating factors from the mesonephros may stimulate the oogonia to enter meiosis and are now called primary oocytes. Recently data has shown that RSPO1/*β*-catenin signaling has a key function in oogonial differentiation by regulating the proliferation and meiosis initiation of the XX germ-cells [19]. Together with pregranulosa cells (follicle cells) they form primordial follicles [20]. 

In the ovary of sheep and bovine fetuses, primary follicles have a diameter of 23 *μ*m to 53 *μ*m. Considering that the diameter of the oocytes varies from 17 *μ*m to 22 *μ*m and that the oogonium has a diameter of 13 *μ*m to 17 *μ*m, it was proposed that the germ cell growing begins before, continues during, and extends after the end of follicular development [4].

The maximum number of nonfollicular germ cells varies from approximately one million in sheep and sows, to several millions in the ovary of cows and women [21]. Near birth and after the degeneration of numerous oocytes during the initial meiotic division, the number of primordial follicles surprisingly decreases at 60% in sows [22] at 80% in rodents, at 90% in women [23], and even more in sheep [24] and cows. Their number is different according to the species, depending directly on the animal's weight [25]. Indeed, the weight of the ovary may have a positive correlation with the number of follicles in young females in good physical condition [26] (Table 1), a fact which may be exploited for biotechnological application in animal husbandry.

 At birth, calves possess approximately 100–150 × 10^3^ follicles. This number probably decreases quite rapidly during postnatal life. A healthy calf possesses 120,000 to 150,000 primordial and primary follicles, 200 to 500 secondary follicles, and 20 to 50 antral follicles [30]. Recently, the presence of a stem-cell population on the ovarian surface has been postulated [31]. This study indicates that germinal stem cells might contribute continuously to the formation of primordial follicles in the adult female. It contradicts the biological paradigm that the follicle pool in mammals which suggests a finite, nonrenewable number. It not only contradicts a long held dogma established by numerous authors that concluded that the pool of primordial follicles decreases in most mammalian species [32, 33]. Besides, the crucial aspect in the development of a functional oocyte is first the onset and conclusion of the first meiotic prophase and second the surrounding of the diplotene oocyte by a string of cells that will form the primordial follicle. It has not been demonstrated in the study of Johnson et al. [31] that either of these two events has taken place. That is why some authors consider it premature to replace the paradigm that neo-oogenesis/folliculogenesis of an adult mammal does not occur [34].

As indicated above, the development of primordial follicles requires the ability of oocytes to detach themselves from the “nests” and associate themselves to precursor cells of the granulosa layer. Oocytes in these nests undergo random apoptosis until they are isolated and then they associate themselves to flat pregranulosa cells to form the primordial follicle [24, 35].

Antral follicles characteristically maintain the meiotic arrest of the oocyte, which depends on high cAMP levels inside the oocyte [36]. The second meiotic division of the oocyte stays arrested during follicular growth and resumes after its release (ovulation) or fertilization. *In vitro*, second meiotic division can continue in a spontaneous way after the oocyte has left the follicle [37], probably due to the absence of the factors responsible for meiotic arrest. These factors are produced by theca cells and are secreted into the follicular liquid. They are called inhibitors of mitotic factors (IMFs), which are polar nonpeptidic molecules that are nonsensitive to heat or to proteolytic enzymes.

The transition from primordial follicle to the stage of primary follicle may be prolonged. While the primordial follicle is characterized by the presence of flat cells (Figure 1), the primary follicles are characterized by a simple layer of cuboid granulosa cells (Figure 1). Preantral follicles have been classified in different ways and numbers, according to the species and authors. Histological studies constituted the basis for the classification to unify the comparison of the factors that may classify these follicles into maturation stages and growth. A simple way as proposed for bovine follicles is the method that classifies follicles according to their size: 30 *μ*m to 90 *μ*m = primordial, primary and small preantral follicles, 90 *μ*m to 150 *μ*m = medium preantrals and 150 *μ*m to 220 *μ*m = large preantrals [2]. A more detailed classification is shown in Table 2.

## 3. Cell Interactions at the Oocyte-Granulosa Cell Interface

The cell-cell and cell-extracellular matrix chemical interactions affect the hormone production and the expression of growth factors of each cell compartment of the follicle (oocyte, granulosa, and theca cells). The interactions also consolidate the differential functions of germinal and somatic lines of the follicle and also coordinate the processes of oogenesis and folliculogenesis [38].

**Figure 2:**
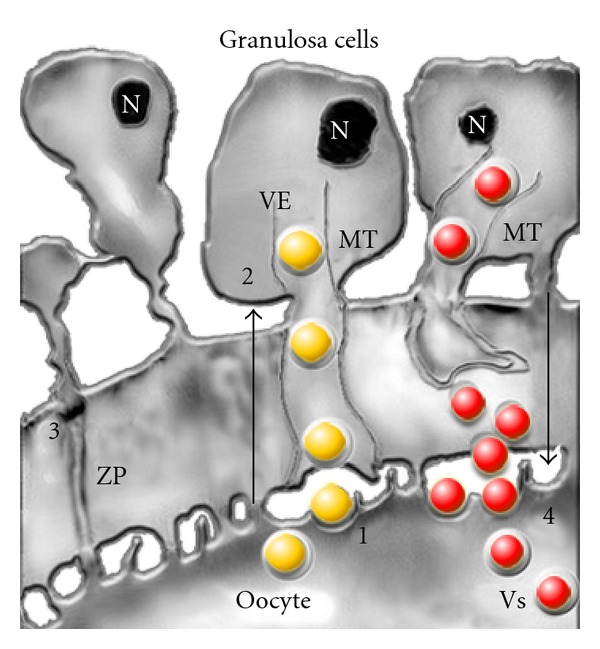
Model proposing regulated delivery of the paracrine factors to the oocyte-granulosa cell interface [6, 7]. Four communication pathways are described. (1) Localized uptake of growth factors, like GDF-9, from the oocyte (yellow ball) by endocytosis at attachment sites of transzonal projections (TZPs) at the oolema. The vectorial transport of endocytic vesicles (VEs) to the cytoplasm of the granulosa cells takes place through the microtubules (MT). (2) Granulosa-zona pellucida interactions are necessary for the orientation of the TZPs. Contact sites may play an important role in the signaling role of oocytes and granulosa cells. (3) The gap junctions that allow direct intercellular communications between the microvilli of the oocyte and the granulosa cell TZPs. (4) Pathway for delivery of granulosa cell factors (red ball) packaged in secretory vesicles (VSs), which undergo endocytosis through the specific receptor on the oocyte surface. N: nucleus, MT: microtubules, ZP: zona pellucida, red ball: factors of the granulosa cells, yellow ball: factors released by the oocyte. With permission from D. F. Albertini.

Since the oocyte development is a critical aspect in the follicular development and the oocyte exerts profound effects on the granulosa cells, the interface between oocyte and granulosa cell emerges as the most significant control site in the coordination of follicle development (Figure 2).

The properties of this interface are considered of fundamental importance for growth regulation and maturation of the oocyte and follicular luteinization [39, 40]. The release of autocrine and paracrine factors then depends directly on the dynamic changes between oocyte and granulosa cells junctions. The reconfiguration of microtubule architecture, in response to stimulation of granulosa cells by means of FSH could lead to retraction of transzonal projections and thus to modulation of the factors released by the oocytes as well as of those released by the granulosa cells [7].

### 3.1. Granulosa Cells

Granulosa cells are shown to be the first cell type in the ovary that provides adequate physical and chemical conditions for oocyte development. In primordial follicles, the female gametes are surrounded by a layer of flattened granulosa cells in an inactivated state [41]. Several factors have been shown to actively suppress follicle activation, including *Sohlh2*, AMH, and *Pten *[42]. *Sohlh* appears important for oogenesis, AMH acts by attenuating the sensitivity of follicles to FHS [43], and Pten represses the phosphatidylinositol 3 kinase (PI3-K) pathway [44, 45]. During folliculogenesis pregranulosa cells differentiate to mature granulosa cells and after ovulation they are transformed into granulosa-lutein cells, which contribute significantly to the corpus luteum. The cells of the cumulus are considered as a subtype of the granulosa cells with morphological and physiological characteristics [46]. Cumulus cells are in permanent contact with the oocyte oolemma. There is also a close contact between the granulosa cells and the theca cells [47]. The granulosa cells adjacent to the oocyte have long cytoplasmic extensions that penetrate the zona pellucida (ZP) and form gap junctions with the oocyte cell membrane. The granulosa cells can therefore contribute to the metabolic processes of development and maturation of the oocytes, which lack some of the molecules necessary for germ-cell growth and metabolism [11, 48].

The regulation of the cytodifferentiation of granulosa cells requires the effect of numerous growth factors and hormones. Besides, there are specific receptors for the gonadotrophic hormones FSH and LH, as well as for factors such as the epidermal growth factor (EGF), insulin like growth factors (IGFs), and the Mullerian inhibiting substance (MIS), also known as anti-Mullerian hormone (AMH), which, according to the stage of differentiation, can be used as a fertility marker [49].

The biosynthesis of hormones like estradiol (E_2_) and progesterone (P) is a primary function of the granulose cells (GCs) in murine, bovine, and human species. As the follicle development continues, the GCs become differentiated and increase the synthesis of E_2_. Another important cell type is the theca cells, which are not present during primordial development but are recruited as nonsteroideogenic precursors during the transition to primary follicle. In contrast to secondary, preantral, and antral follicles, primordial follicle GCs are independent of gonadotropins and steroid hormones [50].The ability of progesterone to prevent oocyte apoptosis is related to the hypothesis that it inhibits the tumor necrosis factor-alpha (TNF*α*) (Table 3, Figure 1), which binds to the cell death receptor. Other studies showed that GCs by themselves were able to maintain the oocyte in meiotic arrest [51].

Another cell factor that can be produced by GCs and that can cause oocyte maturation is the meiosis-activating sterol (FF-MAS) [52] derived from the follicular fluid of female mice and women. The FF-MAS seems to be increased by LH and also by FSH. Although its physiological importance to induce oocyte maturation is controversial, treatment with FF-MAS increased the success rate of maturation in *in vitro* oocyte fertilization of humans [53] and murinae [54].

### 3.2. Cell Bridges or Gap Junctions

**Figure 3:**
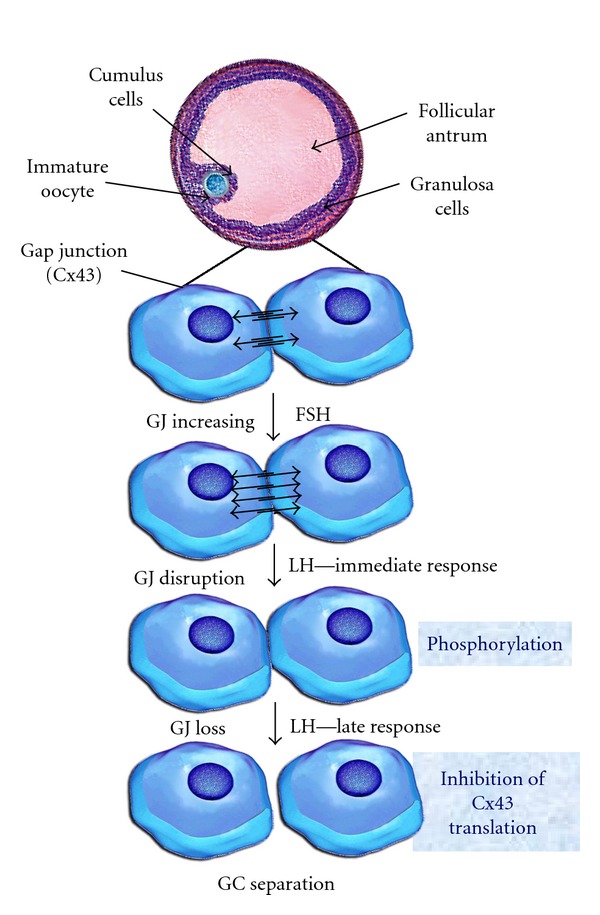
Model of regulation of gap junctional communication (*GJ*, Cx43) [6, 8, 9] During development of the antral follicles FSH hormone stimulates mRNA expression that codifies the Cx43 synthesis of the GJs, the amplification of functional channels, and consequently the integration to the metabolic activity [8]. Mediated by the family mitogen-activated protein kinases, preovulatory LH levels interrupt cell-cell communications by means of phosphorylation and modification of Cx43 protein conformation. This leads to interruption of the intercellular channels. The primary effect of the immediate response to LH is accompanied by elimination of the Cx43 protein, disappearance of GJ sand separation of the GCs from the oocyte. GJ: gap junction; GC: granulosa cells. With permission from N. Dekel.

The cell population of mammalian follicles is strongly interconnected with a net of gap junctions (GJs) [55] that establish a strong exchange of ions, electrical impulses, and small molecules (<1 kD) with the oocyte. The ionic and electrotonic coupling of cells present under the basal membrane, granulosa cells of the cumulus oophorus, and the oocyte create a so-called electrophysiological syncytium. The GJs are regions specialized as transmembrane channels formed by proteins belonging mostly to the connexin family (Figure 3) and are named according to their molecular weight.

The connexin family includes 13 proteins of which Cx26, Cx30.3, Cx32, Cx37, Cx40, Cx43, and Cx45 have been identified in the ovarian tissue of different species [56]. The most abundant one in the follicle is connexin43 (Cx43), present in the granulosa cell channels of the *corona radiata* of bovine oocytes [57] and in the theca. Cx43 is present in primordial and primary follicles of rats and cows and seems to be necessary for the expansion of granulosa cells during follicular development. Cx26 was detected in the connective tissue and blood vessels of sheep and cow oocytes [58, 59]. This connexin may help to maintain the health of the oocyte during follicular growth in cows. However, other studies did not focus on the oocyte-cumulus complex from antral follicles. Cx26 seems to play an important role during CL function and especially during CL regression [59]. Finally, Cx32 is found in cow oocytes and in the luteal and stromal blood vessels [58].

The function of connexins is related to the regulation and coordination of metabolism and cell functions during oocyte development and growth. The GJs were found to facilitate nutrition [60] through transport of molecules from follicle cells to oocytes [61] through the electrical stimuli [62] which summarize the signs of development [63]. Besides, the expression patterns of connexins in the ovary indicate that GJ proteins may play an important role both in the development of the oocyte and in the regulation of folliculogenesis, follicular atresia and development of the luteal body [56] functions that would be regulated hormonally [64]. This characteristic is particularly important for the impermeability of the oocyte membrane to low-weight molecules such as choline, uridine, and inositol [65]. Bidirectional communication between the oocyte and the surrounding granulosa cells allows the transfer of glucose metabolites, amino acids, and nucleotids to the growing oocyte [42]. It has been also hypothesized that an oocyte-granulosa cell regulatory loop may exist that permits the necessary signalling and metabolic pathways to drive growth and development in both compartments [66].

Gonadotropins (FSH and LH) do not participate in the network formation of gap junction protein Cx43 between oocyte and granulosa cells during the development of the primordial and primary follicles. Ultrastructural studies indicated that cell-cell communications in *in vivo *or *in vitro *cultures decrease progressively with time. It is known that the decrease in cell bridges is induced by the increase in LH pulses. This would initiate meiosis resumption caused by reduction of meiosis inhibitory factors flown from the GC to the oocyte by GJs [67]. The progressive decrease in Cx43 protein is also considered a marker of follicular atresia [68]. The removal of the GJs from the oolemma of the oocyte is temporarily correlated with the germinal vesicle rupture or disappearance [57] which confirms the participation of the GJ in the regulation of oocyte meiotic maturation. However, other studies carried out on cattle hypothesized that GVBD takes place before a detectable reduction in the transport between the oocyte and GCs of small radioactively labelled molecules. ^3^H-uridine, Mr 244.2, ^3^H-coline, and Mr 139.5 can be observed [69]. This progressive decrease can be demonstrated until meiosis II (MII).

The cellular bridges for molecules up to 1 kDa may be interrupted when meiotic division starts. However, a second pathway would remain permeable to molecules smaller than 400 Da after the interruption of gap junction communication [57].

The bidirectional communication between oocytes and their cumulus cells by means of GJs seems to be vital for oocyte growth, development, and survival. This interdependency and its persistence are important during oocyte maturation in order to acquire oocyte developmental competence and to facilitate the subsequent embryonic and foetal development [70].

## 4. Autocrine and Paracrine Regulatory Factors

The autocrine and paracrine factors involved in follicle growth and differentiation include proteins and hormones [15, 29]. The ones that regulate the formation of the primordial follicles have not been completely elucidated, although it is known that neurotrophins (NTs) are involved [71] as shown by the presence of 4 of the 5 NTs known. This family of neuronal growth factors (NGFs) is necessary for the survival and differentiation of the neurons in the central and peripheral nervous systems. Besides stimulating and regulating the development of nonneuronal tissues such as those of the immune and cardiovascular systems, NGFs also exhibit great affinity for the ovarian tissue, stimulating the differentiation and development of the mesechymal primordial follicles and granulosa cells as well as synthesis of FSH receptors. Their activity is present even at the time of ovulation, increasing the release of prostaglandin E_2_ (PGE_2_) and the decrease in the communication of gap junctions and stimulation of the theca cell proliferation [71].

 Granulosa cell product Kit ligand (KL), which promotes the function of theca and oocyte cells, induces also Smads 2 and 4 expressions. It also phosphorylates Smads 2 and 4, which are mediators of the transforming growth factor-alpha (TGF*α*), whose function is to regulate follicular growth. Theca cells produce Smad 3 and TGF*α*, keratinocyte growth factor (KGF), and hepatocyte growth factor (HGF). The bone morphogenetic protein-4 (BMP-4), produced by theca and stroma cells, does have critical importance since it promotes the development of the primordial follicle and its transition to primary follicle (Table 3, Figure 1). The Kit ligand (KL) factor, originating from the pregranulosa cells (Figure 1, Table 3), is also known as stem cell factor because it promotes their differentiation, growth and induces the transition from primordial to primary follicle. The basic fibroblast growth factor (bFGF) is located in the oocytes of primordial and primary follicles and influences the development and the transition of primordial follicle by its effect on the granulosa and theca cells. The leukemia inhibitory factor (LIF), released by the pregranulosa and granulosa cells, promotes autocrinally and paracrinally the development of the primordial follicle (Figure 1). The KGF is released by the theca cells and stimulates the transition of the primordial follicle to primary follicle. It is considered to be the first marker that indicates the development of the precursor population of theca cells.

The growth differentiation factor-9 (GDF-9) is an oocyte factor, a member of the TGF-*β* (transforming growth factor-*β*) superfamily that also includes activin and bone morphogenetic proteins (BMPs). The suppression of the GDF-9 gene blocks the development beyond primary follicles [72], decreases cell proliferation of granulosa cells, and causes abnormal oocyte growth [72], which suggests that GDF-9 plays an important role in the development of follicles, probably by upregulating theca cell androgen production [73]. It also prevents apoptosis of granulosa cells. The antiapoptotic effect is so effective that apoptosis begins to be evident at follicles of 200 *μ*m in diameter onwards. GDF-9 has also a positive effect on the on preantral to early antral transition of the follicle and improves blastocyst development [74] and ICM cell numbers [75].

## 5. Diameter of Early Follicles and Oocytes

**Figure 4:**
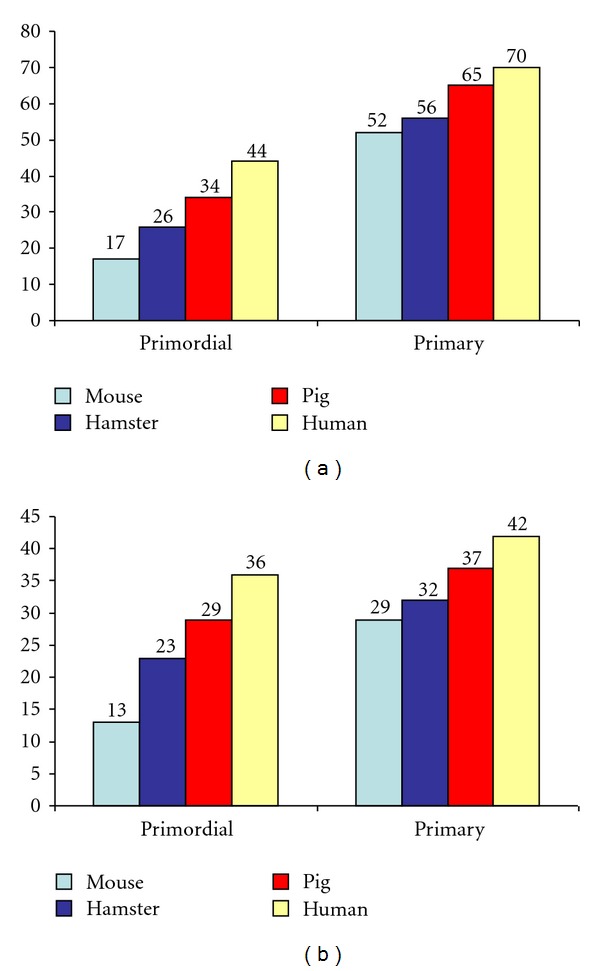
See [6, 10]. (a) Diameters of primordial follicles and primary follicles of 4 species. (b) Oocyte diameters in primordial follicles and primary oocytes of 4 species.

**Figure 5:**
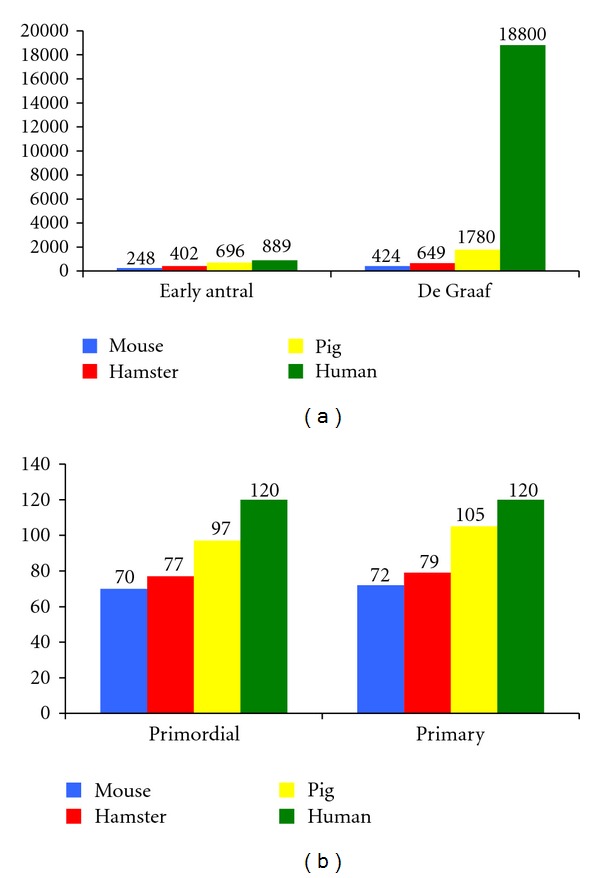
See [6, 10]. (a) Diameters of early antral follicles, and Graafian follicles of 4 species. (b) Oocyte diameters of early antral follicles and Graafian follicles of 4 species.

**Figure 6:**
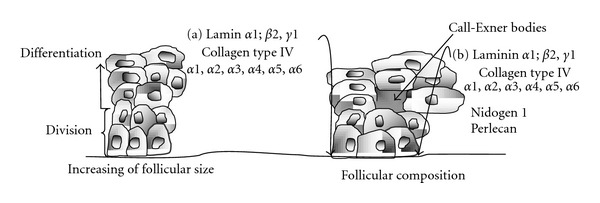
Morphology changes [6, 11, 12] Structure, function and change in the morphology of the granulosa cells [11], components of the basal lamina and the Call-Exner bodies of. (a) Primordial follicles and (b) preantral follicles [12]. With permission from Rodgers [11].

The diameter of the primordial follicles varies between 9 *μ*m and 55 *μ*m and is significantly different among species such as hamster, mouse, pig, and human [10] (Figure 4). The oocyte diameters of the same species, at the same follicular stages, show significant differences in all cases (*P* < 0.005), [10] (Figure 5). Follicular diameters converge during primary (14 *μ*m–62 *μ*m) and preantral stages and then show great disparities during early antral and preovulatory stage when mouse follicles are compared to hamster and pig follicles (Figure 6).

 Bovine and ovine antral follicles of 0.20 mm diameter require approximately 40 days reaching the preovulatory stage. The continuity of their growth depends on a coordinated process of replication and differentiation of their cells and can be divided into two phases. The first, up to a size of 4 mm, depends on the proliferation of granulosa cells and is independent of the hormonal stage. Their development continues to depend on paracrine [76] and endocrine growth factors. The second stage is the one that includes the final development of the follicle until its preovulatory stage. In this process, LH and FSH hormones play a predominant role. Growth factors also exert local modulation of growth antral follicle [76] stimulating proliferation, differentiation [77] and steroidogenesis of follicle wall (Table 4). The fact, that spontaneous apoptosis of the cells was partially suppressed when epidermal growth factor (EGF), Transforming growth factor alpha (TGF-**α**) or Basic fibroblast growth factor (BFGF) were used, corroborates the presence of receptors for those growth factors (Table 4).

Given the fact that FSH was found to induce EGF activity and that FSH in culture medium of preantral hamster follicles prevents atresia [78] it can be hypothesized that at least one mechanism of action of the gonadotrophic hormone could be induced by these growth factors. The growth factor IGF-I (Insulin-like growth factor I) is of critical importance to stimulate the development of the granulosa cells and influences the number of follicles in bovine and porcine species [79]. In mice, deletion of the IGF-I gene leads to failure of ovarian follicles to ovulate because of a block in folliculogenesis at a late preantral or early antral stage [80]. Although the effects of the IGF family (IGF-I-II) and the binding proteins of the insulin-like growth factor are not well understood, the IGF-binding proteins (IGFBPs), depend on species and follicular stage as well as on *in vivo* or *in vitro* developmental conditions [2, 81] only in the late preantral stage starts to grow. IGF-I also has a stimulatory effect on the development of the antral follicles, increasing the sensibility of the granulosa cells to the action of the FSH [81].

The IGFBPs increase the half-life of IGF and thus keep its concentration stable in body tissues. There are 6 IGFBPs (IGFBP1–6) and they have the ability to potentiate as well as to inhibit effects of the IGFs at cellular level.

Oocytes collected from 2–8 mm diameter follicles are covered by cumulus oophorus cells. That is why they are called cumulus-oocyte complexes (COC). *In vivo*, the size of the bovine oocyte is related to the capacity of follicle development. Oocytes competent of development must be at least 110 *μ*m in diameter [82]. The larger the follicle size, the greater the capacity of the oocyte to reach the stage of germinal vesicle breakdown, fertilization, and later development. These abilities are acquired in a sequential way during follicular development [83].

 With the formation of the *antrum*, folds are formed in some species that enlarge the internal surface of the follicle. This surface has no direct relation to the follicle size since the number of layers is very variable [77] or to their morphology [84]. These individual variations, besides the changes in cell shape from columnar to rounded, indicate that the follicle size is not an accurate indication of its development stage [11] which constitutes a limitation in the selection of the follicles either for culture or as a reference in the echographic image for ovum pick up (OPU).

The existence of a spatial progression and differentiation of stem cells located near to the granulosa basal laminato differentiated cells near the follicular *antrum* has been proposed in a similar way as the cell differentiation of the epidermis [11]. However, in the epidermis cell division is limited to basal keratinocytes, whereas cell division in the follicle epithelium is more common in the central areas of the epithelium [85]. Another differential characteristic is the lateral growth of the follicular epithelium during follicular growth, which does not occur in the epidermis. Obviously, granulosa cells can divide and spread laterally without contact inhibition by adjacent cells.

## 6. Genetics of Oocyte Development

The oocyte is an extraordinary and fascinating cell because it can program its nucleus and starts normal embryonic development sometimes even without participation of a spermatozoon (parthenogenesis). Up to now, only a limited number of transcription factors (Table 5) are known to beinvolved in this process. However, recent progress in this field is significant: 90% of the scientific studies focused on the identification and characterization off transcription factors, which play a role in early embryonic development, were published from 2000 onwards. As a result, interesting models appeared proposing a network of gene expression in the murine species as an important experimental model. Studies of most of the factors, shown in Table 5, indicate that many of these transcription factors, which play a role in oocyte and follicle development, are also important for several other important developmental processes.

Oocyte-specific transcription factors expressed in the germinal line and their potential roles in human fertility were extensively studied in murine knockout models (Table 6) [29] and constitute an important line of research at present. Access to the gene list, their definition, and functions may be carried out by OMIM-Online, *Mendelian Inheritance in Man* (PubMed, http://www.ncbi.nlm.nih.gov).

**Figure 7:**
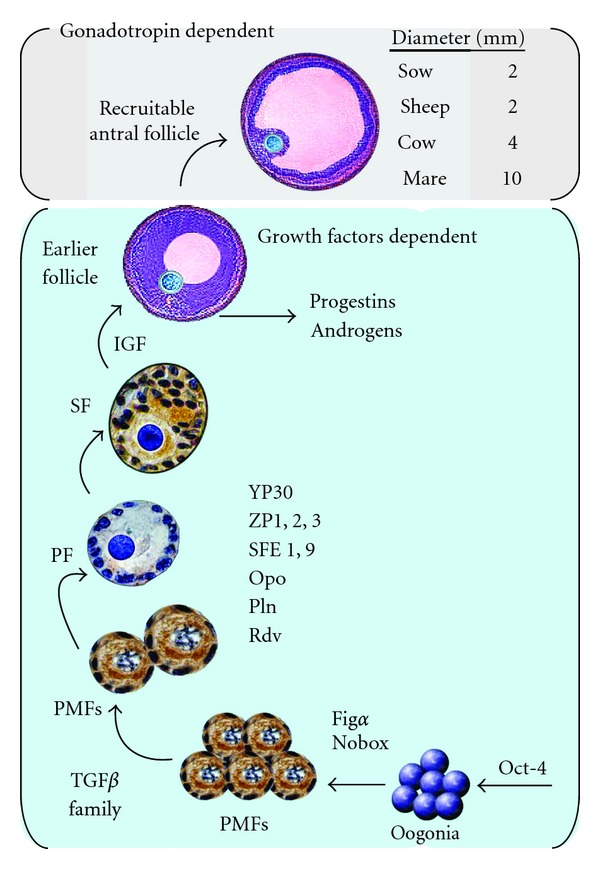
Model of a putative transcription network of gene, protein and hormonal stimulus of follicle development [6, 13, 14]. The process starts with the formation of germ cells and continues with the development of oogonia and oocytes. Several factors are involved in the PGC formation, oogonial (Oct-4), and oocyte development (Fig*α*, Nobox, etc.). Specific oocyte differentiation factors in preantral follicular stages are, YP30, ZP1-3 and those of cortical granulosa content: DF1,9 ovoperoxidase (Opo), proteoliasin (Pln), and rendivin (Rdv). PMFs: Primordial follicles, PF: primary follicle, SF: secondary follicle, IGF (family of the insulin-like growth factors). With permission from Song and Wessel [14].

 Transcription of numerous oocyte genes begins during the early stages of the primordial to primary follicle transition. Follicle and oocyte growth starting at the primordial germ cell stage undergo several transitions. Its onset is marked by differentiation of primordial germ cells, migration of germ cells into the gonadal rudiment, transition of stem cells into primary oocytes, and their maturation. The developmental stages respond to a dynamic network that regulates the progressive transitions of follicle development as well as gene expression factors, [13, 14]. Oogenesis is the process that transforms the proliferative oogonium into an oocyte through meiosis, followed by folliculogenesis and follicular and oocyte maturation. Oogenesis starts with the process of developing oogonia, which occurs via the transformation of primordial follicles into primary oocytes. Oocytogenesis is complete either before or shortly after birth under the effect of several factors, which are involved in the PGC formation, oogonial (Oct-4), and oocyte development (Fig*α*, Nobox, etc.) Figure 7.

## 7. Development of Oocytes inside the Follicles

In bovine and porcine species the capacity to undergo meiosis is acquired more gradually after the formation of the antrum than in mouse [86]. Bovine follicles smaller than 2 mm in diameter show a lower maturation rate and greater susceptibility to fertilization anomalies than larger sized ones [30]. In a similar way, the *in vitro* development of blastocysts was lower when oocytes were obtained from follicles smaller than 6 mm [87].

A similar study comparing follicles of different size (≤3 mm, 3–5 mm and ≥5 mm) confirmed the direct relationship between the size of a follicle and the viability of the oocyte *in vitro* [88]. It is possible that the oocyte acquires its capacity for development during the late follicular growth [88] and that this capacity is not influenced by the beginning follicular atresia. That is the reason why oocytes obtained from atretic follicles possess a capability of development *in vitro* similar to that of developing follicles [89, 90]. Primary oocytes, present in the primordial follicles in meiotic prophase I, result from consecutive mitotic division of oogonia throughout the fetal life. In developing oocytes, meiosis resumption and nuclear maturation in response to gonadotropin stimulation *in vivo* or *in vitro*, is characterized by chromosome condensation, progress from metaphase I to anaphase with extrusion of the first polar body and arrest at metaphase II. In the bovine, prophase of the first meiotic division begins at 70–80 days of fetal life. Primary follicles are made up of the primary oocyte and the surrounding single layer of flat to cuboidal granulosa cells. These cells, precursors of the GC, arrest the oocytes in the dictiyotene stage and prevent the continuation of meiosis. Immature oocytes that have not progressed through meiosis to MII are not able to be successfully fertilized [91].

## 8. Meiotic Arrest

Morphologic classification of MII generally denotes an oocyte that is ‘‘mature,” having arrested in metaphase II, and is presumed to be capable of fertilization. In most mammals meiosis of oocytes is initiated during fetal life but stops prenatally in the dictyotene (diplotene) stage of the prophase I. The meiotic arrest is maintained until follicular development to a Graafian follicle. During that time the oocyte growth adapts itself with that of follicle cells, which interact through the effect of gonadotropin and steroid hormones, as well as with other intrafollicular molecules, such as growth factors.

One of the hypotheses considered for meiotic arrest indicates that the GCs have an inhibitory effect on oocyte maturation. Results obtained by De Loos et al. [92] support that hypothesis because contact interruption of the oocyte with the follicular wall starts the continuation of meiosis [93].

At a diameter of approximately 110 *μ*m in diameter in the bovine, the follicular oocyte reaches the capacity to initiate the meiotic maturation. The size of the oocyte, originating from follicles of 3 mm to 10 mm, varies between 123 *μ*m and 125 *μ*m [94] and has direct relation with the subsequent development rate. Larger sized oocytes possess a greater capacity of development than smaller oocytes [82, 95]. Other follicular factors that can influence the meiotic resumption and subsequent development of oocytes are various components of the bovine follicular fluid (bFF), an exudation of blood plasma modified by the metabolic activity of the follicular wall. It is composed of more than different 40 proteins [96–98], for 13 instance albumin, and lysosomal enzymes, as well as ions [99, 100], ascorbic acid [97, 101–103] steroids (estradiol and progesterone) [97] and gonadotropins (LH and FSH) [104].

Ultimately, it is observed that maturation arrest is a dysfunction on molecular level that results in the incapacity of the oocyte to progress through meiosis either of signalling errors or aberrations in chromosomal/spindle formation [91].

## 9. Conclusion

 Follicular growth during the preantral early antral transition is mainly regulated by intraovarian oocyte-granulosa-theca cell interactions and regulators, such as growth factors, cytokines, and steroids.

The follicle must remain intact in order to preserve its normal function. Actually, there is a sound prospect for the production of molecular tools to classify gamete potential.

Cytokines and growth factors also promote follicular survival and growth during the preantral to early antral transition by suppressing granulosa cell apoptosis and follicular atresia. Thus, for example, GDF-9 enhances preantral follicular growth by upregulating theca cell androgen production.

Assisted reproductive technologies (ARTs) involve controlled follicular development and exogenous hormone stimulation of oocyte maturation. Nevertheless, a successful oocyte *in vitro* maturation (IVM) would eliminate the need for hormonal stimulation used in ART. In addition, by understanding the molecular and cellular mechanisms in the control of follicular development during the preantral-to-early antral transition, it is possible that IVM protocols could be developed to provide the signalling machinery necessary for oocyte maturation competence.

This should be facilitated by gene expression data exchange and translation into a better understanding of the underlying biological phenomena.

## Figures and Tables

**Table 1:** Oocyte loss during meiosis [15].

Species	Maximum number of oocytes	Number of oocytes at birth	Oocyte loss (%)
Murine	50,000–75,000	10,000–15,000	80
Ovine	900,000	82,000	91
Porcine	1,200,000	500,000	58
Bovine	2,700,000	135,000	95
Human	7,000,000	700,000	90

**Table 2:** Classification and characterization of bovine follicles [2, 27].

Follicle	No. of GC layers	No. of GC	Diameter of follicle (*μ*m)	Diameter of oocyte (*μ*m)	ZP	Inner theca
Primordial (type 1)	1	<10 flat	<40	30	No	No
Primary (type 2)	1–1.5	10–40 cuboidal	40–80	31	No	No
Small preantral (type 3)	2-3	41–100	81–130	49	No	No
Large preantral (type 4)	4–6	101–250	131–250	69	+	+
Small antral (type 5)	>6	>250	250–500	93	++	++

**Table 3:** Factors that can promote growth and development of preantral follicles [2, 3].

Factor	Origin	Function
TNF*α*	Oocyte	Regulates apoptosis of oocytes
BMP15	Oocyte	Differentiation and proliferation of primary follicles
GDF 9	Oocyte	Differentiation and proliferation of primary follicles
EGF/bFGF	Oocyte	Induces development of primordial and preantral follicles
Fig*α* (Figla)	Oocyte	Coordination of structural genes for development of the primordial follicle such as Zp1, Zp2, and Zp3 of the ZP
Protein ckit	Oocyte and theca	Transition of primordial follicle to primary follicle. Migration and proliferation of germ cells
Activin	Granulosa cells	Proliferation of granulosa cells
KL	Pregranulosa cells	Induces development of primordial follicle to primary follicle
GH	Endocrine	Development of GC and theca cells of preantral follicles
Insulin	Endocrine	Induces development of the primordial follicle to primary follicle
IGF-I	Granulosa cells	Stimulates development from preantral follicle to antral follicle
AMH	Granulosa cells	Inhibition of primordial follicle development
AhR	Granulosa cells	Regulation of the oocyte pool size
BMP4	Theca/stroma	Promotes transition from primordial follicle to primary follicle and oocyte survival
KGF	Theca	Proliferation of granulosa cells

AhR: Aromatic hydrocarbon receptor, AMH: anti-Mullerian hormone, BMP: bone morphogenic protein, EGF/bFGF: epidermis growth factor/basic fibroblast growth factor, GDF: growth differentiation factor, GH: growth hormone, Fig*α*: factor in the germline alpha, IGF: insulin growth factor, KGF: keratinocyte growth factor, KL: Kit ligand, TNF: tumor necrosis factor.

**Table 4:** Localization of EGF, TGF-*α*, EGF-R proteins and mRNA in the bovine ovary [28].

Cell type	EGF	EGF-r	TGF-*α*	EGF		EGF-r		TGF-*α*	
				Prot.	RNA	Prot.	RNA	Prot.	RNA
Oocyte	+	+	+	+	—	+	(+)	+	(+)
Cumulus	+	+	+	+	(+)	+	+	+	(+)
Granulosa	+	+	+	+	(+)	+	+	+	+
Theca	—	—	+	—	—	—	+	+	+

	Preantral follicles			Antral follicles					

EGF: epidermal growth factor, EGF-r: EGF receptor; TGF-*α*: transforming growth factor alpha, +: yes; —: no; Prot.: proteins.

**Table 5:** Transcription factors related to follicular and oocyte development [14].

Factor	mRNA expression		Protein localization	
	Oocytes	Ovary	Oocyte	Ovary
Oct-4	+++	—	+++	—
Fig*α*	+++	—	+++	—
NoBox	+++	—	+++	—
TAFII 105	—	+++	n/d	n/d
FOXL2	+	+++	—	+++
Oogenesin 1	+++	—	+++	—
Sox 3	+	++	—	—
Obox 1,2,3,4,5,6	++	—	n/d	n/d

—: not detected; n/d: not determined.

**Table 6:** Murine model of transcription factors expressed in the gonads and their possible effect on fertility [29].

Expressed preferentially in germ cells		
Gene	Number	Mouse knockout phenotype
*Fig*α**	Factor in the germline alpha	Infertility, oocyte loss at postnatal day 2
*Nobox (Og2x)*	Newborn ovary homebox	Infertility, oocyte loss at postnatal day 14, disrupted primordial to primary transition
*Oct4 (Pou5fl)*	POU domain, class 5, transcription factor	Maintenance of primordial germ cells
*Taf4b *(*TAFIII05*)	TATA box binding protein-associated factor 4b	Infertility, folliculogenesis blocked at preantral stage
Expressed in the granulosa and somatic cells of the ovary		
*Foxo3a*	Forkhead box O3a	Initially fertile, infertility at 15 weeks, progressive loss of viable follicles, with total loss at 18 weeks
*Fox12*	Forkhead box L2	Infertility, block at the primordial and primary follicle stage
*Sox3*	Sry-box containing gene 3	Infertile mice with normal follicular development, unclear defect
*Sf1 *(*Nr5a1*)	Nuclear receptor subfamily 5, group A, member 1	1. conditional knock out infertile, ovary develops, antral follicles form, absent corpora lutea
*Lrh-1 *(*Nr5a2*)	Nuclear receptor subfamily 5, group A, member 2	Mice die by *E*6.5–*E*7.5
*Wt1*	Wilms tumor homologue	Gonads degenerate by *E*12.5, similar to *Sf1 *
*Lhx9*	LIM homeobox protein 9	Gonads fail to proliferate by *E*11.5, primordial germ cell migration unaffected
*Emx2*	Empty spiracles homologue 2	Mice die soon after birth and lack kidneys, ureters and gonads. Primordial germ cells migrate normally
